# Technostress Among University Teachers in Higher Education: A Study Using Multidimensional Person-Environment Misfit Theory

**DOI:** 10.3389/fpsyg.2019.01791

**Published:** 2019-08-06

**Authors:** Xinghua Wang, Bo Li

**Affiliations:** ^1^Normal College, Qingdao University, Qingdao, China; ^2^Center for Research and Development in Learning, Nanyang Technological University, Singapore, Singapore; ^3^Institute of Higher Education, Linyi University, Linyi, China

**Keywords:** technostress, multidimensional person-environment misfit, higher education, university teachers, information and communication technologies

## Abstract

To investigate the phenomenon of technostress among university teachers in higher education, a multidimensional person-environment misfit framework of technostress was proposed and validated by 343 teachers from universities in China. The findings indicate that person-organization (P-O) misfit, person-technology (P-T) misfit, and person-people (P-P) misfit largely captured how university teachers interact with multiple dimensions of the higher education environment in an imbalanced way that causes technostress. P-O misfit predicted P-T misfit and P-P misfit. Relationships between multidimensional technostress and job performance were investigated. It was found that university requirements related to the use of ICT and the suitability of ICT for university teachers’ work were critical factors affecting their job performance. In addition, a comparison was made among university teachers from different grade levels, revealing that university management related to ICT use tended to affect university teachers of higher-grade levels more than those of lower-grade levels in generating technostress.

## Introduction

Universities worldwide have been advancing their agendas for education enhanced by information and communication technologies (ICT), such as promoting mobile learning, blended learning, and virtual reality-based instruction (Markowitz et al., 2018; Qi, 2019). Admittedly, these efforts are laudable and could potentially benefit learners. However, they could also exert increased pressure on university teachers who are often less technology-savvy than their students (Jena, 2015; Hatlevik and Hatlevik, 2018), but have to constantly adapt themselves to the ever-demanding university requirements related to the use of technologies at work, which is exacerbated by the rapid changes and advancement of ICT. As a result, the incongruence or misfit between universities and teachers may lead to the latter being subjected to technostress, which is defined as a modern maladaptation resulting from the failure to cope with ICT and changing requirements related to the use of ICT (Brod, 1984; Fuglseth and Sørebø, 2014).

Taking the perspective of person-environment (P-E) fit theory, which has been essential in technostress research (Edwards et al., 1998; Ayyagari et al., 2011), technostress is basically the consequence of misfit between a person and the environment surrounding the person. Given that the person-environment misfit has multiple dimensions (Chuang et al., 2016), technostress is not only related to the ICT that causes it, but it is also related to organizations that set requirements on the use of ICT (Tarafdar et al., 2010) and colleagues within organizations who often have influence on individuals’ use of ICT (Avanzi et al., 2018). Consequently, technostress caused by misfits between teachers and multiple dimensions of the university environment could negatively affect university teachers’ performance at work, leading to job burnout and even intentions of quitting the job (Al-Fudail and Mellar, 2008; Tarafdar et al., 2015; Pignata et al., 2016).

In spite of negative consequences associated with technostress to university teachers’ work, extant research on technostress has primarily been focused on government and business sectors (e.g., Ayyagari et al., 2011; Fuglseth and Sørebø, 2014). Limited research has investigated this issue in education, particularly in higher education settings where university teachers are exposed to varied ICT and rapid advancement of ICT is revolutionizing learning and teaching (Jena, 2015; Ortagus et al., 2018). Moreover, prior research investigating technostress from the P-E fit perspective mostly examined single dimensions of the environment such as organizations or jobs within organizations (e.g., Player et al., 2017), thus producing an incomplete understanding of technostress and subsequently impeding the development of informed solutions of this issue.

In view of the context-dependent characteristic of technostress (Tarafdar et al., 2015) and the multidimensional nature of the environment (Jansen and Kristof-Brown, 2006), it is imperative to examine technostress in higher education from a more comprehensive perspective. Therefore, this study aimed to bridge this gap by developing a multidimensional person-environment misfit framework of technostress, taking into consideration multiple dimensions of the university environment where university teachers are situated.

## Theoretical Framework

### The Paradox of ICT

The use of ICT in organizations has been fraught with controversy and paradoxes (Tarafdar et al., 2011). With higher education being gradually digitalized, there is little doubt that ICT bring benefits to university teachers’ work. ICT such as mobile computing, collaborative software, and learning management system enable teachers to work from anywhere and anytime, access information conveniently, and upgrade their teaching (Jena, 2015; Qi, 2019). On the other hand, ICT also presents challenges to people’s physical and psychological well-being and their job performance (Ayyagari et al., 2011). For instance, ICT may drive university teachers to work faster than they can sustain (techno-overload) and invade their personal life (techno-invasion). Frequent changes and upgrades of software and hardware often make university teachers feel incompetent (techno-complexity and techno-uncertainty). Moreover, fast technological advances such as Massive Online Open Course (MOOCs) may threaten their job security (techno-insecurity). Consequently, university teachers may feel exhausted, anxious, and stressful. This phenomenon is termed as technostress and will likely affect university teachers’ job effectiveness (Al-Fudail and Mellar, 2008; Jena, 2015).

### Extant Research on Technostress

Technostress, as a dark side of ICT, is a relatively new and understudied area, in contrast to considerable numbers of topics on benefits associated with ICT to people’ work and life (Tarafdar et al., 2010). Currently, research on technostress has been conducted mostly in government and business environments (e.g., Ragu-Nathan et al., 2008; Fuglseth and Sørebø, 2014). Despite the limited number of studies on technostress in the field of education (e.g., Al-Fudail and Mellar, 2008; Joo et al., 2016), the prevalence and severity of this issue in this field, particularly in higher education, may be no less pronounced than that in government and business environments, in view of the huge investment on ambitious agendas of modernizing learning and teaching via ICT (Glenn, 2008).

Nevertheless, there has been a consensus regarding possible consequences of technostress across different fields. Technostress could negatively affect people’s productivity and innovation in their tasks, leading to decreased job performance, lower job satisfaction, and higher turnover rates (Ayyagari et al., 2011; Tarafdar et al., 2015). As such, the issue of technostress deserves proper attention from researchers, developers of ICT, and policy-makers in organizations, including higher education institutions.

Prior studies investigating technostress have been done mainly from two perspectives: transaction theory of stress (e.g., Fuglseth and Sørebø, 2014) and person-environment fit (e.g., Ayyagari et al., 2011).

Transaction theory of stress describes the issue of stress as a combination of stimulating conditions and individuals’ responses to them (Lazarus and Folkman, 1984). According to this theory, the emergence of technostress seems to be a linear process, from stressors and situational factors to strain and outcomes. Studies based on this theory have mostly followed a reductionist approach in which technostress creators and inhibitors are differentiated and singled out to examine this issue (e.g., Ragu-Nathan et al., 2008).

P-E fit theory highlights the extent to which a person and the environment match (Edwards et al., 1998). Compared with the transactional-based approach, P-E fit theory acknowledges the complex characteristics of technostress. It argues that stress neither arises from the person nor the environment alone; instead, it emerges from the interaction of both. In other words, technostress arises when misfit between the person and the environment happens. But, studies following P-E fit theory have often examined the fit/misfit between the person and a single aspect of the environment (Jansen and Kristof-Brown, 2006), such as either organizations, people, or jobs. This is inconsistent with the fact that people are concurrently nested in multiple dimensions of the environment (Chuang et al., 2016).

As a result, studies examining technostress from a single dimension of P-E misfit or treating its emergence as a linear process may generate a limited understanding of technostress and its impact on individuals’ job performance and psychological well-being. Therefore, this study argues for a multidimensional P-E misfit framework to investigate the phenomenon of technostress in higher education. This study proposes that the formation of technostress results from the unbalanced interaction between the person (teacher) and multiple dimensions of the university environment, and that different dimensions of P-E misfits have influence on one another.

### Multidimensional P-E Misfit Framework of Technostress

Contrasting to the conventional P-E fit approach which often focuses on a single aspect of the environment, the multidimensional P-E fit theory highlights the importance of multiple characteristics of the environment (Jansen and Kristof-Brown, 2006). In line with the multidimensional P-E fit theory and the context of this study, we considered three dimensions of P-E fit: person-organization (P-O) fit, person-technology (P-T) fit, and person-people (P-P) fit. “Organizations” (i.e., universities) in P-O fit refers to the management of universities, including demands for teachers to meet university goals such as requirements and regulations, as well as resources available for university teachers to meet these demands, such as technical support, training, and culture (Demerouti et al., 2001; Avanzi et al., 2018). “Technologies” in P-T fit are concerned with varied ICT employed by universities to digitalize teaching, research, and faculty management. “People” in P-P fit refers to colleagues of teachers in universities in this study as supervisors and managers who are often policymakers in universities are subsumed under “organizations” in P-O fit.

Based on the definition of P-E fit theory and research on multidimensional P-E (mis)fit, P-O fit, P-T fit, and P-P fit are defined as the extent to which the person fits organizations, technologies, and colleagues, respectively. Technostress arises when misfits between the person and the multiple dimensions of the environment occur. In line with the above reasoning, P-O misfit, P-T misfit, and P-P misfit form the multidimensional P-E misfit framework of technostress.

While we can analyze technostress as misfits between a person and multiple dimensions of the environment, another important factor is the causes of the misfits. According to Edwards et al. (1998), stress emerges when (a) the environment does not provide sufficient supplies to meet the person’s needs; and/or (b) the person’s abilities do not meet the environment’s demands. As such, a P-E misfit is often investigated in two ways: *abilities-demands (A-D) misfit and/or needs-supplies (N-S) misfit* (Player et al., 2017).

In addition, P-E fit theory distinguishes from general interactionist models of the person and the environment in that P-E fit theory requires both the person and the environment constructs to be commensurate with each other (Edwards et al., 1998). For instance, A-D misfit on the organizational level (P-O misfit) should involve the comparison between the amount of ICT use in teaching demanded by universities and the amount of ICT use teachers could incorporate in their teaching. Accordingly, *abilities-demands and need-supplies* in the multidimensional P-E misfit framework of technostress in this study’s context are defined in the following ways:

#### Demands of Organizations and Technologies Versus Abilities of the Person

Demands refer to quantitative and qualitative job requirements from universities related to technological use (*the organizational level*) and requirements of ICT for effective use of them (*the technological level*). Abilities are concerned with university teachers’ skills, aptitudes, and time to meet demands from universities and ICT. When demands exceed university teachers’ abilities, the misfit is likely to yield technostress. However, as to *the people level* misfit in the multidimensional P-E misfit framework, we only focused on N-S interaction as this dimension of misfit only investigated social support from colleagues in the use of ICT at work.

#### Needs of the Person Versus Supplies by Organizations, Technologies, and Colleagues

“Needs” broadly refers to teachers’ requirements for universities to support effective use of ICT (*the organizational level*), teachers’ requirements for ICT to assist them in fulfilling job objectives (*the technological level*), and teachers’ requirements for colleagues to help them effectively use ICT at work (*the people level*).

Supplies are related to resources and support provided by universities to enable teachers to effectively integrate ICT into their work, functions of ICT available in helping teachers achieve job objectives, and social support from colleagues in stimulating effective ICT use. When supplies fall short of university teachers’ needs, the misfit tends to generate technostress.

### Operationalization of Multidimensional P-E Misfit Framework of Technostress

In this study, we propose a nested structure by operationalizing the emergence of technostress (A-D misfit and N-S misfit) in the multiple dimensions of person-environment interactions (see Table 1), as explained below.

**TABLE 1:** Nested structure of multidimensional P-E misfit framework of technostress.

	**Peron-organization (P-O) misfit**		**Person-technology (P-T) misfit**		**Person-people (P-P) misfit**
**Operationalizing each**	**Abilities-demands**	**Needs-supplies**	**Abilities-demands**	**Needs-supplies**	**Needs-supplies**
**misfit dimension**	**(A-D) misfit**	**(N-S) misfit**	**(A-D) misfit**	**(N-S) misfit**	**(N-S) misfit**
Acronyms of combinations	ADO	NSO	ADT	NST	PPT

#### A-D Misfit in P-O Misfit (ADO)

The implementation of ICT in universities increases requirements of job scope and skills for university teachers, who are likely to experience higher task difficulty and more ambiguity about performance expectations from universities (Ragu-Nathan et al., 2008), thereby tending to create an incongruence or misfit between abilities of teachers and demands of universities.

#### N-S Misfit in P-O Misfit (NSO)

As integrating ICT in teaching often necessitates new and higher skills from university teachers and changes of work processes (Tarafdar et al., 2010), university support is essential in preparing teachers to adapt to the changes. Support, such as technical support and professional training, is considered important in helping university teachers integrate ICT into their work, while insufficient university support is likely to intensify teachers’ stress during ICT integration (Joo et al., 2016).

#### A-D Misfit in P-T Misfit (ADT)

Due to constant updates of ICT hardware and software and their increasing complexities, university teachers’ skills are subject to be devaluated frequently. In addition, university teachers may also feel inundated by vast amounts of information from multiple ICT such as learning management systems, social media, and staff management systems. They are compelled to work faster to deal with increased processing demands, consequently creating a gap between abilities of teachers and demands of ICT for a better use of them (Hogan and McKnight, 2007; Ragu-Nathan et al., 2008).

#### N-S Misfit in P-T Misfit (NST)

ICT often needs to be reconfigured and customized before being applied to university teachers’ daily work. However, modifications of ICT often lead to problems such as system crashes, data loss, and inadequate technical resources (Ragu-Nathan et al., 2008). Consequently, the ICT to be used may fall short of meeting university teachers’ needs. Furthermore, because of the lack of teachers’ involvement during ICT purchase and implementation phases, the ICT may turn out to be unable to match teachers’ work requirements (Tarafdar et al., 2010). This not only causes unnecessary financial waste, but also negatively affects university teachers’ job performance.

#### P-P Misfit (PPF)

Person-people misfit is mainly concerned with the lack of support from colleagues in the use of ICT at work. Colleagues’ social support constitutes an important resource to deal with technostress at work (Avanzi et al., 2018). It can induce positive affect among university teachers, as they perceive that they do not cope with high work demands alone. However, the lack of colleagues’ social support tends to accentuate people’s feelings of helplessness, especially in the face of work challenges (Al-Fudail and Mellar, 2008).

### Hypothesis Development

Overall, this study was guided by the following two research questions: (a) What are the relationships among the multidimensional P-E misfits of technostress? and (b) How do the multidimensional P-E misfits of technostress affect university teachers’ job performance? To help answer the research questions, the hypotheses related to the multidimensional P-E misfits of technostress and job performance would be developed.

Organizational management related to ICT directly determines what ICT are needed and how ICT are used in organizations (Tarafdar et al., 2010). Inappropriate organizational management may cause the introduction of unsuitable ICT and/or improper implementation of ICT (Al-Fudail and Mellar, 2008), which may end up either being too complex for teachers to handle or being insufficient to satisfy their needs for teaching.

Organizational management also affects working relationships among university teachers in organizations (Hayton et al., 2012). Effective organizational management can foster prosocial behaviors and increase teamwork, thus increasing social support among university teachers (Avanzi et al., 2018). However, improper organizational management likely distances university teachers from one another and decreases their chances of obtaining sufficient help from colleagues to cope with challenges at work (Luchman and González-Morales, 2013). Thus, we hypothesized that P-O misfit (containing ADO and NSO) is fundamental to P-T misfit (containing ADT and NST) and P-P misfit in the present study. As such, the following hypotheses were developed:

H1. Abilities-demands misfit on the organizational level (ADO) positively predicts abilities-demands misfit on the technological level (ADT).
H2. Abilities-demands misfit on the organizational level (ADO) positively predicts needs-supplies misfit on the technological level (NST).
H3. Abilities-demands misfit on the organizational level (ADO) positively predicts person-people misfit (PPF).
H4. Needs-supplies misfit on the organizational level (NSO) positively predicts abilities-demands misfit on the technological level (ADT).
H5. Needs-supplies misfit on the organizational level (NSO) positively predicts needs-supplies misfit on the technological level (NST).
H6. Needs-supplies misfit on the organizational level (NSO) positively predicts person-people misfit (PPF).

Job performance in this study refers to the degree to which ICT helps university teachers complete their job requirements (Tarafdar et al., 2010). As ICT gradually becomes an integrated part of organizations including higher education institutions, they have an increasing influence on university teachers’ performance and the success of organizations. Furthermore, university teachers’ job performance plays a significant role in preparing students for the future workforce and is essential in maintaining universities’ competitiveness in the global education market (Jena, 2015).

Based on extant research (Tarafdar et al., 2011; Chuang et al., 2016), the fit between university teachers and multiple dimensions of the higher education environment leads to improved job performance, while the misfit results in otherwise. Understanding how university teachers’ job performance is affected by the multiple dimensions of the environment is important in addressing technostress in higher education and in better utilizing benefits associated with ICT. Therefore, we hypothesized that the multidimensional P-E misfits of technostress negatively affect university teachers’ job performance.

H7. Abilities-demands misfit on the organizational level (ADO) negatively predicts job performance.
H8. Needs-supplies misfit on the organizational level (NSO) negatively predicts job performance.
H9. Abilities-demands misfit on the technological level (ADT) negatively predicts job performance.
H10. Needs-supplies misfit on the technological level (NST) negatively predicts job performance.
H11. Person-people misfit (PPF) negatively predicts job performance.

**FIGURE 1:**
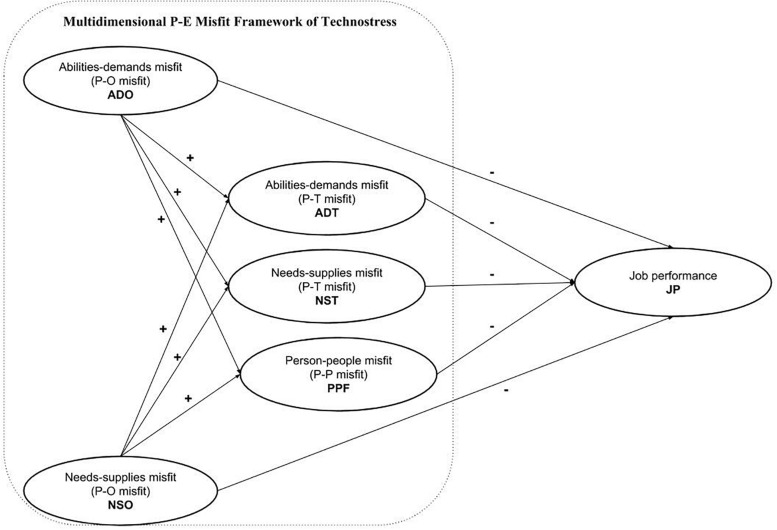
The proposed research model for this study.

Based on the analyses above, the research model of this study was developed and is illustrated in Figure 1.

## Methodology

### Participants

The participants of the study were sampled from five public universities in mainland China. To respond to the call from the Minister of Education (MOE) of China to modernize learning and teaching via ICT (MOE of PRC, 2015), the five public universities, among many others in the country, have been advancing agendas such as digitalizing curricula via MOOC, promoting mobile learning, and experimenting with ICT-enhanced flipped classrooms. To accomplish the agendas, teachers in the universities are required to learn to create video and audio teaching resources of different lengths, utilize learning management systems for routine work, and adjust their teaching practices in some courses toward learner-centered and ICT-enhanced pedagogy. Consequently, many teachers in the universities complain about the technostress generated as a result of these agendas.

To facilitate the implementation of these agendas in the public universities in the country, the MOE sponsored a series of professional development programs aiming to increase university teachers’ capabilities of ICT-enhanced learning and teaching. The participants of this study who were from the five public universities were assigned in one of the programs. Among the 400 participants approached, with their informed consent valid responses from 343 participants were obtained, with a response rate of 85.75%. Their demographic information is presented in Table 2.

**TABLE 2:** Demographic information of the teacher participants (*N* = 343).

		***N***	**Percentage**
Age	26–30 years	37	10.79
	31–40 years	222	64.72
	41–50 years	63	18.37
	51–60 years	18	5.25
	61 years and above	3	0.87
Gender	Male	114	33.24
	Female	229	66.76
Grade levels of teaching	Year 1	89	25.95
	Year 2	118	34.40
	Year 3	105	30.61
	Year 4	20	5.83
	Postgraduate schools	11	3.21
Total participants		343	

### Instrument Development and Validation

The initial survey instrument contained six constructs with 37 items (see Appendix A) and was rated on a five-point Likert scale, with 1 representing *Strongly Disagree* and 5 *Strongly Agree* with the items.

In the initial survey, 31 items were developed to operationalize multidimensional P-E misfits of technostress, comprising of ADO, NSO, ADT, NST, and PPF, based on Edwards et al. (1998), Jansen and Kristof-Brown (2006), Chuang et al. (2016). Specifically, abilities-demand misfit on the organizational level (ADO) was originally described by eight items, for instance, “*I find it difficult to meet the high demands of school policies regarding the use of ICT at work*” and “*I find it hard to adjust my current work pattern so as to comply with school policies regarding the use of ICT at work.*” Needs-supplies misfit on the organizational level (NSO) contained five items, for example, “*My school does not provide me with sufficient professional training to effectively use ICT at work*,” and “*My school does not provide me with sufficient incentives to effectively use ICT at work.*” Abilities-demands misfit on the technological level (ADT) had seven items, such as “*I find it difficult to effectively use ICT due to my limited investment of time and effort*,” and “*I find it difficult to cope with the high demands of ICT with my current capability.*” Needs-supplies misfit on the technological level (NST) contained seven items, for instance, “*The ICT in my school are not effective in helping me increase my productivity at work*,” and “*The ICT in my school are not very relevant for the improvement of my work*.” Person-people misfit (PPF) was described by four items, such as “*I do not have sufficient support from my colleagues for the use of ICT at work*,” and “*My colleagues are not encouraging with regard to the innovative use of ICT at work*.”

The construct of job performance was adapted from Tarafdar et al. (2010) and contained six items, such as “*The ICT in my school improve the quality of my work*” and “*The ICT in my school enhance my work productivity*.” The reported Cronbach’s alpha of job performance was 0.91.

To ensure the clarity of the original survey items, three participants were invited to check their understanding and the wording of the survey, which was subsequently reworded to deliver clearer ideas related to technostress. Furthermore, to improve the face validity of the original survey, two experts in stress research were approached to obtain their comments on the survey items, which was refined accordingly. In addition, because the original survey was in English, we conducted a back-translation to ensure that there was minimal difference between the English and Chinese versions.

Given that the five constructs (ADO, NSO, ADT, NST, and PPF) measuring multidimensional P-E misfits of technostress were self-developed, it was necessary to examine the internal consistency and validity of this technostress model before proceeding to further analyses. A number of rounds of exploratory factor analysis (EFA) were first performed on the technostress model consisting of the five constructs to extract the preliminary factor structure, which was then examined via confirmatory factor analysis (CFA; see Appendix B for detailed criteria of performing EFA and CFA and related results). In the end, the refined technostress model comprising the five constructs with 22 items was attained. Cronbach’s alpha values for ADO, NSO, ADT, NST, and PPF were 0.90, 0.89, 0.79, 0.86, and 0.86, respectively. The refined technostress model obtained good model fit (χ^2^*/df* = 2.06, CFI = 0.95, NFI = 0.91, and RMSEA = 0.06).

Given that this study utilized self-report data, Harman’s single-factor test (Podsakoff et al., 2012) was carried out to examine possible common method bias. After entering all variables into an EFA to investigate the unrotated factor solution, the total variance explained by a single factor was 33.29%, which was considerably lower than 50%. This suggests that there is no significant amount of common method bias existing in the data.

### Data Analysis

PLS-SEM was used to analyze the research model as illustrated in Figure 1. As a variance-based structural equation modeling, PLS-SEM is prediction-oriented and exploratory with the aim of maximizing the variance explained for the dependent variables (Chin, 1998; Hair et al., 2014). This study’s use of PLS-SEM was mainly based on the following two reasons. First, PLS-SEM works effectively with small sample sizes. Because the PLS-SEM algorithm analyzes portions of the PLS-SEM model one by one and iteratively, it is viable if the sample size is sufficient to calculate the single largest regression equation in the model rather than the whole model (Willaby et al., 2015). Second, PLS-SEM is good at prediction and theory development. It can generate stronger or equivalent statistical power than covariance-based structural equation modeling with smaller sample sizes (Reinartz et al., 2009; Astrachan et al., 2014). Considering that this study endeavored to develop a multidimensional P-E misfit framework of technostress and is exploratory in nature, PLS-SEM fits well with the aim of the present study. The PLS-SEM package (Sanchez, 2013) in the R programing language was used for data analysis.

## Results

The research model in this study was analyzed via PLS-SEM following two steps: the measurement model and the structural model (Hair et al., 2014). Then, the whole dataset was split based on university teachers’ grade levels as higher-grade courses have more complicated knowledge structures and those teaching higher-grade courses are often senior teachers, thus being more likely to be subjected to technostress (McIver et al., 2016). Subsequently, multi-group comparisons were carried out on the sub-datasets to examine whether there were any possible differences regarding technostress among university teachers of different grade levels, the knowledge of which could inform the development of targeted countermeasures against technostress.

Considering that we categorized five grade levels of teaching (Year 1, Year 2, Year 3, Year 4, and Postgraduate schools) and there were very unequal numbers of participants in different grade levels, we combined participants of Year 1 and Year 2 and labeled them as Lower-grade levels (*N* = 207). Participants of Year 3, Year 4, and Postgraduate studies were added up and labeled as Higher-grade levels (*N* = 136).

### Measurement Model

The measurement model of this study was investigated from the following criteria: (a) item reliability, (b) convergent validity, and (c) discriminant validity.

#### Item Reliability

Item reliability is assessed by examining the loadings of survey items with their respective latent construct. The standardized loadings of the items should exceed 0.70 (Hair et al., 2014). Table 3 indicates that the loadings of all items satisfied the requirement.

**TABLE 3:** Cronbach’s alpha, composite reliability, average variance extracted (AVE), and factor loadings of the constructs and items in the research model (*N* = 343).

**Constructs/ Items**	**Cronbach’s alpha**	**Composite reliability**	**AVE**	**Factor loadings**	***M (SD)***
Abilities-demands misfit (in P-O misfit)	0.90	0.92	0.71		
ADO1				0.85	2.83 (1.11)
ADO2				0.84	2.87 (1.14)
ADO3				0.86	2.67 (1.15)
ADO4				0.83	2.84 (1.16)
ADO5				0.82	2.85 (1.13)
Needs-supplies misfit (in P-O misfit)	0.89	0.93	0.76		
NSO1				0.84	3.64 (1.14)
NSO2				0.85	3.85 (1.09)
NSO3				0.90	3.68 (1.10)
NSO4				0.90	3.83 (1.08)
Abilities-demands misfit (in P-T misfit)	0.79	0.87	0.62		
ADT1				0.72	3.48 (1.17)
ADT2				0.77	3.32 (1.20)
ADT3				0.82	2.86 (1.19)
ADT4				0.82	2.91 (1.21)
Needs-supplies misfit (in P-T misfit)	0.86	0.90	0.64		
NST1				0.72	2.82 (1.15)
NST2				0.76	2.69 (1.17)
NST3				0.87	2.84 (1.28)
NST4				0.80	2.94 (1.23)
NST5				0.83	2.69 (1.21)
Person-people misfit (P-P misfit)	0.86	0.90	0.70		
PPF1				0.82	3.27 (1.18)
PPF2				0.85	3.23 (1.12)
PPF3				0.84	3.66 (1.12)
PPF4				0.82	3.43 (1.17)
Job performance	0.95	0.96	0.79		
JP1				0.90	3.58 (1.00)
JP2				0.93	3.54 (1.01)
JP3				0.92	3.64 (0.99)
JP4				0.84	3.54 (1.02)
JP5				0.87	3.71 (0.94)
JP6				0.87	3.77 (0.93)

#### Convergent Validity

This criterion investigates the extent to which survey items that are theoretically related to one another are related in practice (Hair et al., 2011). Convergent validity is examined by checking (a) internal consistency and (b) average variance extracted (AVE) of each latent construct (Fornell and Larcker, 1981). Internal consistency of a given latent construct is assessed through composite reliability. An internally consistent model should have composite reliability of above 0.70 (Nunnally, 1978). As shown in Table 3, the research model met the requirement. As to AVE, the minimum AVE value of 0.50 suggests that at least 50% of the variance of the indicators is explained (Hair et al., 2011). As indicated in Table 3, AVEs of the latent constructs in the research model satisfied the requirement. Taken together, the convergent validity of the research model in this study was substantiated.

#### Discriminant Validity

The discriminant validity of the research model was examined in two aspects: (a) the square root of the AVE for each latent construct shall exceed the correlation coefficients between that and other latent constructs (Chin, 1998); and (b) survey items should load more on the latent constructs that they aim to measure than on other latent constructs (Chin et al., 2003). As shown in Table 4 and Appendix C, the two requirements of discriminant validity were supported.

**TABLE 4:** Discriminant validity of the research model (*N* = 343).

**Constructs**	**ADO**	**NSO**	**ADT**	**NST**	**PPF**	**JP**
Abilities-demands misfit (in P-O misfit) ADO	**0.84**					
Needs-supplies misfit (in P-O misfit) NSO	0.27	**0.87**				
Abilities-demands misfit (in P-T misfit) ADT	0.63	0.23	**0.79**			
Needs-supplies misfit (in P-T misfit) NST	0.67	0.31	0.52	**0.80**		
Person-people misfit (P-P misfit) PPF	0.51	0.51	0.42	0.44	**0.83**	
Job performance JP	–0.31	–0.07	–0.14	–0.38	–0.11	**0**.**89**

*The bold values in the diagonal row are the square roots of the average variance extracted for the constructs in the research model.*

### Structural Model

**FIGURE 2:**
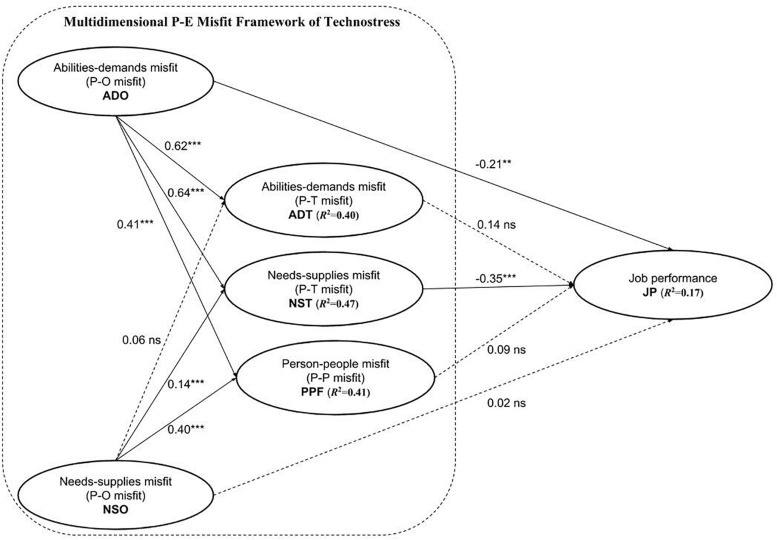
The validated structural model (*N* = 343). ^∗∗^*p* < 0.01; ^∗∗^*p* < 0.001; ns = nonsignificant.

This study’s structural model was assessed through path coefficients’ significance levels and explanatory power (i.e., *R*^2^) of endogenous constructs. Figure 2 illustrates the validation outcomes of the structural model in this study. Bootstrapping analyses were utilized to examine the statistical significance of the path coefficients in the structural model as PLS-SEM does not rely on distributional assumptions and thus the significance levels are not suitable to be examined through parametric approaches (Hair et al., 2014).

Table 5 presents the bootstrapping validation outcomes. Overall, P-E misfit on the organizational level (P-O misfit: ADO and NSO) largely served as the fundamental misfit, underlying the functioning of P-E misfit on the technological (P-T misfit: ADT and NST) and people levels (P-P misfit). Specifically, ADO significantly predicted ADT, NST, and PPF, thereby supporting H1, H2, and H3. NSO significantly predicted NST and PPF, except for ADT; thus, H5 and H6 were substantiated while H4 was not. With regards to the effect of multidimensional P-E misfits of technostress on university teachers’ job performance, ADO and NST demonstrated strong negative effects. As such, H7 and H10 were supported. However, NSO, ADT, and PPF did not exert significantly negative effects on job performance; therefore, H8, H9, and H11 were not supported.

**TABLE 5:** Bootstrap validation outcomes for the research model (*N* = 343).

**Hypotheses**		**Path coefficients**	**Results**
**H1**	**ADO - > ADT**	**0.62^∗∗∗^**	**Support**
**H2**	**ADO - > NST**	**0.64^∗∗∗^**	**Support**
**H3**	**ADO - > PPF**	**0.41^∗∗∗^**	**Support**
H4	NSO - > ADT	0.06	Not support
**H5**	**NSO - > NST**	**0.14^∗∗∗^**	**Support**
**H6**	**NSO - > PPF**	**0.40^∗∗∗^**	**Support**
**H7**	**ADO - > JP**	−**0.21^∗∗∗^**	**Support**
H8	NSO - > JP	0.02	Not support
H9	ADT - > JP^#^	0.14	Not support
**H10**	**NST - > JP**	−**0.35^∗∗∗^**	**Support**
H11	PPF - > JP	0.09	Not support

*^∗∗^*p* < 0.01; ^∗∗∗^*p* < 0.001; ADO, abilities-demands misfit (in P-O misfit); NSO, needs-supplies misfit (in P-O misfit); ADT, abilities-demands misfit (in P-T misfit); NST, needs-supplies misfit (in P-T misfit); PPF, person-people misfit (P-P misfit); JP, job performance; #The bootstrap analysis did not support the statistical significance of the path coefficient (see Appendix D) due to the possible risk of overfitting issue; The bold rows highlight the hypotheses that were supported in this study.*

As PLS-SEM aims to maximize the variance explained in endogenous constructs, *R*^2^ values of endogenous constructs are viewed as the primary criterion for assessing the quality of structural models (Henseler et al., 2009). However, due to the lack of generally agreed-upon values of *R*^2^, this study followed the research of Cohen (1988) on R2. Cohen (1988) pointed out that *R*^2^ values of 0.02, 0.13, and 0.26 imply small, medium, and large effect sizes, respectively. As shown in Figure 2, the *R*^2^ values of ADT, NST, and PPF were 0.40, 0.47, and 0.41, respectively, indicating substantial explanatory power. The *R*^2^ value of JP was 0.17, implying a moderate explanatory power. On the whole, the predictive power of this study’s model was acceptable.

A global criterion of goodness-of-fit (0 < GoF < 1) has been developed by Tenenhaus et al. (2004) to assess the overall quality of PLS-SEM analyses. It is computed as the geometric mean of the average communality and average *R*^2^. The GoF values of 0.10, 0.25, and 0.36 are defined as small, medium, and large, respectively (Wetzels et al., 2009). The research model’s GoF value in this study was 0.51, which was considerably large. In sum, the reliability and validity of the proposed research model in this study were confirmed and acceptable according to the analyses above.

### Multi-Group Comparison Based on Grade Levels of Teaching

Multi-group comparison is conducted by comparing differences at structural levels of research models. Specifically, path coefficients of research models based on different groups of participants are examined (Sanchez, 2013; Hair et al., 2014), as the aim of path modeling with latent constructs is to estimate linear relationships among the constructs. The approach of bootstrap *t-*test was used by following a three-step procedure: (a) the whole dataset is split into groups; (b) bootstrap samples are carried out with replacements for each group; (c) subsamples are compared through *t*-tests in terms of standard error estimates of path coefficients.

Table 6 reveals that there were significant differences between university teachers of different grade levels regarding (a) *the path coefficient of ADO on ADT*, indicating that ADO exerted a greater influence on ADT for university teachers of higher-grade levels than those of lower-grade levels; and (b) *the path coefficient of NSO on PPF*, implying that NSO had a more significant effect on PPF for teachers of higher-grade levels than those of lower-grade levels.

**TABLE 6:** Comparison between teachers of lower- and higher-grades (*N* = 343).

**Path**	**Global**	**Group: lower**	**Group: higher**	***diff.abs***	***t***	***df***	***p***	**Sig.05**
**ADO - > ADT**	**0.62**	**0.55**	**0.71**	**0**.**16**	**1**.**88**	**341**	**0.03**	**Yes**
ADO - > NST	0.64	0.59	0.70	0.11	1.41	341	0.08	No
ADO - > PPF	0.41	0.47	0.33	0.14	1.33	341	0.09	No
NSO - > ADT^#^	0.06	0.15	–0.09	0.24	2.90	341	0.002	No
NSO - > NST	0.14	0.16	0.11	0.05	0.77	341	0.22	No
**NSO - > PPF**	**0.40**	**0.32**	**0.53**	**0**.**21**	**1**.**74**	**341**	**0.04**	**Yes**
ADO - > JP	–0.21	–0.24	–0.15	0.09	0.53	341	0.30	No
NSO - > JP	0.02	–0.04	0.12	0.16	1.25	341	0.11	No
ADT - > JP^#^	0.14	0.22	–0.02	0.23	1.82	341	0.03	No
NST - > JP	–0.35	–0.40	–0.32	0.08	0.65	341	0.26	No
PPF - > JP^#^	0.09	0.19	–0.10	0.29	1.96	341	0.03	No

*ADO, abilities-demands misfit (in P-O misfit); NSO, needs-supplies misfit (in P-O misfit); ADT, abilities-demands misfit (in P-T misfit); NST, needs-supplies misfit (in P-T misfit); PPF, Person-people misfit (P-P misfit); JP, job performance; diff.abs, absolute difference; #The comparisons make sense only when the global path coefficients are statistically significant; The bold row indicates the paths where teachers of lower-grades significantly differed from those of higher-grades.*

## Discussion and Conclusion

This study investigated the phenomenon of technostress among university teachers in higher education. A research model containing a multidimensional P-E misfit framework of technostress was proposed to examine technostress from a more comprehensive perspective: person-organization (P-O) misfit, person-technology (P-T) misfit, and person-people (P-P) misfit, and how the multidimensional P-E misfits of technostress negatively affected university teachers’ job performance.

The findings indicate that the proposed research model demonstrated high reliability and validity. P-O misfit basically played a fundamental role, greatly predicting P-T misfit [with the *R*^2^ values of 0.40 and 0.47 for abilities-demands misfit (ADT) and needs-supplies misfit on the technological level (NST), respectively] and P-P misfit [with the *R*^2^ value of 0.41 for person-people misfit (PPF); see Figure 2]. This implies that organizational management, including organizational demands of ICT use and supplies to university teachers to meet the demands, largely determines the emergence of technostress and thus, is essential in preserving university teachers’ well-being. In this regard, although technostress is associated with technologies in the first place, it is more likely to be caused by organizational management related to technological use.

Moreover, among the five constructs [abilities-demands misfit on the organizational level (ADO), needs-supplies misfit on the organizational level (NSO), abilities-demands misfit on the technological level (ADT), needs-supplies on the technological level (NST), and person-people misfit (PPF)] in the framework of technostress, ADO (with a path coefficient of −0.21) and NST (with a path coefficient of −0.35; see Table 5) had a significantly negative influence on university teachers’ job performance. This suggests that, when keeping university teachers’ abilities and needs constant, organizational demands of ICT use and suitability of ICT for teachers’ work are crucial factors in determining the effectiveness of teachers’ work.

With regards to the comparison between university teachers of lower and higher grades, ADO had a more significant effect on ADT for university teachers of higher-grade than those of lower-grade; NSO also exerted a greater influence on PPF for university teachers of higher-grade than those of lower-grade. This implies that for higher-grade university teachers, the impact of organizational management on other dimensional misfits is more significant, thereby contributing more to technostress.

For the sake of delivering coherent themes based on the statistical findings, hypotheses of this study are better discussed collectively, instead of one by one. Overall, four themes were generated, as follows:

(1) P-O misfit, P-T misfit, and P-P misfit may largely capture how university teachers interact with the multiple dimensions of the higher education environment in imbalanced ways that generate technostress, with P-O misfit underlying the functioning of the other two misfits.

The validated multidimensional P-E misfit framework of technostress suggests that the use of P-O (person-organization) misfit, P-T (person-technology) misfit, and P-P (person-people) misfit to describe the formation of technostress is largely aligned with incongruent interactions between university teachers and the multiple dimensions of the higher education environment (Chuang et al., 2016). Although technostress is a type of stress related to ICT, it is also associated with universities that require ICT-enhanced learning and teaching (Ragu-Nathan et al., 2008), as teachers themselves usually do not have sufficient motivation to volunteer to change their established teaching practices by integrating ICT (Joo et al., 2016).

Furthermore, it is also associated with the availability of support from colleagues in universities, which is considered a coping strategy and one of the most important resources for employees to deal with work stress (Halbesleben, 2006). Insufficient support from colleagues may cause great stress when job demands are high (Pignata et al., 2016; Avanzi et al., 2018), as it decreases university teachers’ confidence and abilities to cope with challenges related to the use of ICT.

In addition, universities are structured organizations with a clear hierarchy of management and collective goals, as have been identified in other occupational settings (Avanzi et al., 2018). Thus, the fundamental role played by P-O misfit in triggering P-T misfit and P-P misfit is largely in line with the characteristic of universities as a form of organization, as evidenced by Hypotheses 1–6 except for H4 (see Figure 2 and Table 5).

Finally, with regard to the effect size of job performance (*R*^2^ = 0.17) in the whole research model, it is understandable that the multidimensional P-E misfits of technostress negatively contributed a moderate amount of variance in job performance. Although ICT are considered essential to modernization agendas of many universities and play an increasingly important role in teachers’ work, they have not yet dominated teachers’ work. As such, the *R*^2^ value of 0.17 of job performance was already sufficient to consider technostress hazardous.

(2) Requirements of universities related to ICT use at work are critical in affecting university teachers’ job performance.

As shown in Figure 2 and Table 5, within the multidimensional P-E misfits, ADO (abilities-demands misfit on the organizational level) was found to exert a significantly negative influence on university teachers’ job performance (H7) while ADT (abilities-demands misfit on the technological level) did not (H9). This may be due to the fact that while being able to use ICT is one thing, being able to satisfy universities’ requirements related to ICT use at work may be another. This is because ICT-enhanced learning, such as flipped classroom, mobile learning, and blended learning, involves new teaching paradigms and philosophies which require changes in university teachers’ roles in classroom, content delivery, pedagogical design, and assessment (Hogan and McKnight, 2007), instead of simply possessing or applying ICT knowledge. Consequently, teachers may struggle to meet universities’ requirements of integrating ICT into work in spite of their mastery of ICT.

However, despite the fundamental role played by P-O (person-organization) misfit in the multidimensional P-E misfit framework, NSO (needs-supplies misfit on the organizational level) in P-O misfit failed to predict ADT (abilities-demands misfit on the technological level; H4) and did not significantly jeopardize university teachers’ job performance (H8). These findings about NSO may seem surprising, as previous studies consider university support imperative in preparing teachers to integrate ICT into work (e.g., Al-Fudail and Mellar, 2008; Luchman and González-Morales, 2013). This may be due to the fact that teachers often avoid university supplies such as professional development workshops and technical seminars as they do not want to be overwhelmed by various computing requirements or made to feel ignorant (Shedletsky and Aitken, 2001). Moreover, the ICT-enhanced learning paradigm often consumes more time and effort than onsite teaching (Hogan and McKnight, 2007; Joo et al., 2016) and has few relations to university teachers’ promotion, tenure, and salary (Glenn, 2008). Consequently, teachers tend to perceive the university supplies limitedly relevant to their career development (Shedletsky and Aitken, 2001). Therefore, even if needs-supplies misfit occurs on the organizational level (NSO), university teachers’ job performance is not likely to be significantly compromised. Likewise, whether university teachers are capable of utilizing ICT has little relevance to NSO.

(3) Suitability of ICT for university teachers’ work is significantly important to their job performance.

As revealed in Figure 2 and Table 5, NST in P-T (needs-supplies misfit on the technological level) misfit exerted a significantly negative effect on university teachers’ job performance (H10), while ADT (abilities-demands misfit on the technological level) and PPF (person-people misfit) did not (H9 and H11). This may be because university teachers in higher education are often professionals in their fields and tend to have higher capabilities in developing their ICT knowledge and skills (Tarafdar et al., 2011). Even if acquiring mastery of ICT involves steep learning curves and/or sufficient social support from colleagues is not available, teachers could spend more time and effort to master the ICT by themselves. As a result, even if ADT and PPF may affect university teachers’ work (Tarafdar et al., 2010; Avanzi et al., 2018), they are not likely to significantly undermine their job performance. Nevertheless, once the ICT available to university teachers cannot satisfy their work needs, the resultant needs-supplies misfit on the technological level (NST) would easily cause a significantly negative effect on university teachers’ job performance, regardless of how capable teachers are. Specifically, the ICT should have necessary functional properties that are suitable for and support university teachers’ work, instead of setting limits for their work. For instance, if a learning management system stipulates a variety of requirements of formats and properties of teaching resources that can be uploaded, it will restrict university teachers’ choices of course design and implementation, eventually negatively affecting their job performance.

(4) University teachers teaching higher-grade classes are more likely to be significantly affected by universities’ requirements and supplies related to the use of ICT.

The comparison outcomes between university teachers of lower and higher grades (see Table 6) can be discussed from characteristics of senior and junior teachers, as grade levels of teaching often represent different levels of skillsets and abilities; in the context of this study, senior teachers often teach and supervise higher-grade students.

While ICT are revolutionizing education of all kinds, policy-makers in educational systems are placing increasing levels of pressure on teachers to digitalize their work (Orlando, 2014). Senior teachers who have taken years in establishing their teaching practices are under greater burdens to change them than their younger counterparts who are mostly in the process of developing their teaching practices (Day and Gu, 2009; Orlando, 2014). Consequently, senior teachers may find it more difficult to cope with the integration of ICT into their work.

As organizational supplies such as professional training and technical support are often of general types and apply to every university teacher (Shedletsky and Aitken, 2001), they may be eventually insufficient and improper for teachers of higher grades who need specialized training programs and support to digitalize their established traditional teaching practices. In addition, higher-grade courses often involve more complex knowledge structures (McIver et al., 2016). Thus, integrating ICT into higher-grade courses tend to involve greater workloads and may be more challenging for these teachers. As a result, the unsupportive work environment is more likely to reduce senior teachers’ possibilities of attaining their desired help from colleagues to deal with challenges of adjusting to the ICT-enhanced learning paradigm (Luchman and González-Morales, 2013), thereby leading to higher possibilities of P-P misfit.

### Contributions

This study has the following contributions. First, this study developed a multidimensional P-E misfit framework to investigate technostress among university teachers in higher education. The multidimensional P-E misfits consist of P-O misfit, P-T misfit, and P-P misfit, among which P-O misfit holds a fundamental position and underlies the functioning of the other two dimensions of misfits. In view of the dearth of studies on technostress in higher education settings and the reductionist approach utilized by extant studies in examining this issue, the multidimensional P-E misfit framework of technostress provides a more comprehensive theoretical perspective in understanding the formation of technostress.

Second, to facilitate future research on technostress in higher education, this study also designed and validated an instrument to measure technostress from multiple dimensions. The instrument has demonstrated high psychometric properties and can be utilized in future theoretical and empirical efforts for fuller knowledge of technostress. In the finalized multidimensional P-E misfit scale of technostress with 22 items (the total score ranging from 22 to 110), the higher the score, the greater the levels of technostress. Specifically, a score of 22 indicates the absence of technostress; 23–65 corresponds to a mild level of technostress; 66–87 implies a moderate level of technostress; and ≥ 88 corresponds to a severe level of technostress.

Third, this study revealed differences among university teachers from different grade levels in the causes of technostress through the multidimensional P-E misfit framework, thus highlighting possible differences between senior and junior teachers in their experiences of technostress and possibilities of suffering from technostress due to varying workloads associated with integrating ICT in different grade levels of courses.

Fourth, among the A-D and N-S misfits in the multidimensional P-E misfit framework of technostress, the A-D misfit on the organizational level (ADO) and the N-S misfit on the technological level (NST) were identified to have significantly negative effects on university teachers’ job performance. Therefore, these findings could effectively inform the development of targeted strategies for addressing the issue of technostress among teachers in higher education.

### Implications

This study carries the following implications for future research and practice on technostress in higher education. First, in the multidimensional P-E misfit framework developed in this study, organizational management plays an important and fundamental role in the formation of technostress, which is more likely to arise when organizational demands are high and organizational supplies are insufficient (Griffiths, 2014). Organizational management largely determines what ICT should be introduced, how ICT should be implemented, and the availability of support university teachers may get from colleagues (Tarafdar et al., 2010; Avanzi et al., 2018). In addition, ADO and NST exerted significant negative effects on university teachers’ job performance. Therefore, the priority of developing countermeasures against technostress should start from adjusting university management related to the use of ICT.

Universities with cases of teachers reporting the issue of technostress should reconsider their primary objectives of ICT use and align university objectives with ICT affordances and teachers’ real needs related to ICT. Teachers should be involved in ICT planning, purchase, and implementation phases (Tarafdar et al., 2010). Universities can identify teachers’ real needs of ICT for their work if teachers are allowed to participate in discussions related to ICT adoption. Also, incorporating teachers’ real needs and suggestions into universities’ decision-making from the beginning can help improve the match between university demands and what teachers can provide and between teachers’ needs and what ICT can provide, thereby decreasing possible technostress yielding from the mismatches and improving university teachers’ job performance.

Second, as university teachers of higher-grade levels are more likely to be negatively affected by improper organizational management related to ICT use, universities advancing their digitization agendas should not adopt the one-size-fits-all strategies. Instead, considering that university teachers of higher-grade levels have often spent years developing their work practices and higher-grade courses often involve more complex knowledge structures, university demands of ICT use for them should be modest and university supplies should be tailored specifically to their characteristics.

Third, as technostress is basically a psychological reaction (Ragu-Nathan et al., 2008), it is imperative that teachers themselves learn to regulate their emotional and psychological responses to external challenges. More importantly, they should also seek to develop their capabilities and skills to effectively cope with challenges associated with ICT-enhanced learning and teaching paradigm.

### Limitations and Future Research

Certain limitations should be considered when interpreting this study’s findings. First, this study adopted a cross-sectional design, thus making it difficult to attain causal relationships among constructs related to technostress. To strengthen the explanatory power of the multidimensional P-E misfit framework of technostress, a longitudinal design is necessary in future research. Second, in view of possible influence of different cultures on technostress, the generalization of the study’s findings should be cautious. Future research is suggested to validate the multidimensional P-E misfit framework of technostress in Western universities to obtain a more comprehensive understanding of technostress in higher education. Third, the participants of this study may not optimally represent university teachers in higher education, as the number of female participants doubles that of males. Researchers in the future are advised to balance the gender distribution of participants to further validate the findings of this study. Fourth, this study’s findings were attained through self-report data, thereby subjecting to report errors related to human perceptions. As such, future studies may consider triangulating self-report data with possible clinical diagnostic tests of technostress and in-depth interviews with university teachers, administers, and other stakeholders in higher education to provide stronger arguments for the phenomenon of technostress and inform the development of solution strategies.

## Data Availability

The datasets generated for this study are available on request to the corresponding author.

## Ethics Statement

This study was conducted in accordance with the recommendations of the Institutional Review Board (IRB), Nanyang Technological University with written informed consent from all participants.

## Author Contributions

XW designed the study, analyzed the data, and wrote the manuscript. BL contributed to the design of the study and data collection.

## Conflict of Interest Statement

The authors declare that the research was conducted in the absence of any commercial or financial relationships that could be construed as a potential conflict of interest.

## References

[B1] Al-Fudail M. Mellar H. (2008). Investigating teacher stress when using technology. *Comput. Educ.* 51 1103–1110. 10.1016/j.compedu.2007.11.004

[B2] Astrachan C. B. Patel V. K. Wanzenried G. (2014). A comparative study of CB-SEM and PLS-SEM for theory development in family firm research. *J. Fam. Bus. Strategy* 5 116–128. 10.1016/j.jfbs.2013.12.002

[B3] Avanzi L. Fraccaroli F. Castelli L. Marcionetti J. Crescentini A. Balducci C. (2018). How to mobilize social support against workload and burnout: the role of organizational identification. *Teach. Teacher Educ.* 69 154–167. 10.1016/j.tate.2017.10.001

[B4] Ayyagari R. Grover V. Purvis R. (2011). Technostress: technological antecedents and implications. *MIS Q.* 35 831–858. 10.2307/41409963

[B5] Brod C. (1984). *Technostress: The Human Cost of The Computer Revolution.* Boston, MA: Addison Wesley Publishing Company.

[B6] Chin W. W. (1998). “The partial least squares approach for structural equation modeling,” in *Methodology for Business and Management. Modern Methods for Business Research*, ed. Marcoulides G. A. (Mahwah, NJ: Lawrence Erlbaum Associates Publishers), 295–336.

[B7] Chin W. W. Marcolin B. L. Newsted P. R. (2003). A partial least squares latent variable modeling approach for measuring interaction effects: results from a monte carlo simulation study and an electronic-mail emotion/adoption study. *Inform. Syst. Res.* 14 189–217. 10.1287/isre.14.2.189.16018

[B8] Chuang A. Shen C. T. Judge T. A. (2016). Development of a multidimensional instrument of person–environment fit: the perceived person–environment fit scale (PPEFS). *Appl. Psychol.* 65 66–98. 10.1111/apps.12036

[B9] Cohen J. (1988). *Statistical Power Analysis for The Behavioral Sciences*, 2nd Edn Hillside, NJ: Lawrence Earlbaum Associates.

[B10] Day C. Gu Q. (2009). Veteran teachers: commitment, resilience and quality retention. *Teacher Teach.* 15 441–457. 10.1080/13540600903057211

[B11] Demerouti E. Bakker A. B. Nachreiner F. Schaufeli W. B. (2001). The job demands-resources model of burnout. *J. Appl. Psychol.* 86 499–512. 10.1037/0021-9010.86.3.499 11419809

[B12] Edwards J. R. Caplan R. D. Harrison V. R. (1998). “Person-environment fit theory: conceptual foundations, empirical evidence, and directions for future research,” in *Theories of Organizational Stress*, ed. Cooper C. L. (Oxford: Oxford University Press), 28–67.

[B13] Fornell C. Larcker D. F. (1981). Structural equation models with unobservable variables and measurement error: algebra and statistics. *J. Mark. Res.* 18 382–388. 10.1177/002224378101800313

[B14] Fuglseth A. M. Sørebø Ø (2014). The effects of technostress within the context of employee use of ICT. *Comput. Hum. Behav.* 40 161–170. 10.1016/j.chb.2014.07.040

[B15] Glenn M. (2008). The future of higher education: how technology will shape learning. Avaliable at: https://www.learntechlib.org/p/182088/ (accessed October 20, 2008).

[B16] Griffiths A. (2014). Promoting resilience in schools: a view from occupational health psychology. *Teacher Teach.* 20 655–666. 10.1080/13540602.2014.937954

[B17] Hair J. F. Hult G. T. M. Ringle C. Sarstedt M. (2014). *A Primer on Partial Least Squares Structural Equation Modeling (PLS-SEM).* Thousand Oaks: Sage.

[B18] Hair J. F. Ringle C. M. Sarstedt M. (2011). PLS-SEM: indeed a silver bullet. *J. Mark. Res. Theor. Pract.* 19 139–152. 10.2753/MTP1069-6679190202

[B19] Halbesleben J. R. (2006). Sources of social support and burnout: a meta-analytic test of the conservation of resources model. *J. Appl. Psychol.* 91 1134–1145. 10.1037/0021-9010.91.5.1134 16953774

[B20] Hatlevik I. K. Hatlevik O. E. (2018). Examining the relationship between teachers’ ICT self-efficacy for educational purposes, collegial collaboration, lack of facilitation and the use of ICT in teaching practice. *Front. Psychol.* 9:935. 10.3389/fpsyg.2018.00935 29951018 PMC6008425

[B21] Hayton J. C. Carnabuci G. Eisenberger R. (2012). With a little help from my colleagues: a social embeddedness approach to perceived organizational support. *J. Organ. Behav.* 33 235–249. 10.1002/job.755

[B22] Henseler J. Ringle C. M. Sinkovics R. R. (2009). “The use of partial least squares path modeling in international marketing,” in *Advances in International Marketing*, eds Sinkovics R. R. Ghauri P. N. (Bingley: Emerald Group Publishing Limited), 277–319. 10.1108/s1474-7979(2009)0000020014

[B23] Hogan R. L. McKnight M. A. (2007). Exploring burnout among university online instructors: an initial investigation. *Internet High. Educ.* 10 117–124. 10.1016/j.iheduc.2007.03.001

[B24] Jansen K. J. Kristof-Brown A. (2006). Toward a multidimensional theory of person-environment fit. *J. Manag.* 18 193–212.

[B25] Jena R. (2015). Technostress in ICT enabled collaborative learning environment: an empirical study among Indian academician. *Comput. Hum. Behav.* 51 1116–1123. 10.1016/j.chb.2015.03.020

[B26] Joo Y. J. Lim K. Y. Kim N. H. (2016). The effects of secondary teachers’ technostress on the intention to use technology in South Korea. *Comput. Educ.* 95 114–122. 10.1016/j.compedu.2015.12.004

[B27] Lazarus R. S. Folkman S. (1984). *Stress, Sppraisal, and Coping.* New York, NY: Springer.

[B28] Luchman J. N. González-Morales M. G. (2013). Demands, control, and support: a meta-analytic review of work characteristics interrelationships. *J. Occup. Health Psychol.* 18 37–52. 10.1037/a0030541 23339747

[B29] Markowitz D. M. Laha R. Perone B. P. Pea R. D. Bailenson J. N. (2018). Immersive virtual reality field trips facilitate learning about climate change. *Front. Psychol.* 9:2364. 10.3389/fpsyg.2018.02364 30555387 PMC6284182

[B30] McIver D. Fitzsimmons S. Flanagan D. (2016). Instructional design as knowledge management: a knowledge-in-practice approach to choosing instructional methods. *J. Manag. Educ.* 40 47–75. 10.1177/1052562915587583

[B31] Nunnally J. (1978). *Psychometric Methods.* New York, NY: McGraw-Hill.

[B32] Orlando J. (2014). Veteran teachers and technology: change fatigue and knowledge insecurity influence practice. *Teacher Teach.* 20 427–439. 10.1080/13540602.2014.881644

[B33] Ortagus J. C. Kramer D. A. Umbricht M. R. (2018). Exploring the IT productivity paradox in higher education: the influence of IT funding on institutional productivity. *J. High Educ.* 89 129–152. 10.1080/00221546.2017.1341756

[B34] Pignata S. Winefield A. H. Provis C. Boyd C. M. (2016). Awareness of stress-reduction interventions on work attitudes: the impact of tenure and staff group in Australian universities. *Front. Psychol.* 7:1225. 10.3389/fpsyg.2016.01225 27588011 PMC4988981

[B35] Player D. Youngs P. Perrone F. Grogan E. (2017). How principal leadership and person-job fit are associated with teacher mobility and attrition. *Teach. Teacher Educ.* 67 330–339. 10.1016/j.tate.2017.06.017

[B36] Podsakoff P. M. MacKenzie S. B. Podsakoff N. P. (2012). Sources of method bias in social science research and recommendations on how to control it. *Annu. Rev. Psychol.* 63 539–569. 10.1146/annurev-psych-120710-100452 21838546

[B37] Qi C. (2019). A double-edged sword? Exploring the impact of students’ academic usage of mobile devices on technostress and academic performance. *Behav. Inform. Technol.* 1–18. 10.1080/0144929X.2019.1585476

[B38] Ragu-Nathan T. Tarafdar M. Ragu-Nathan B. S. Tu Q. (2008). The consequences of technostress for end users in organizations: conceptual development and empirical validation. *Inform. Syst. Res.* 19 417–433. 10.1287/isre.1070.0165

[B39] Reinartz W. Haenlein M. Henseler J. (2009). An empirical comparison of the efficacy of covariance-based and variance-based SEM. *Int. J. Res. Mark.* 26 332–344. 10.1016/j.ijresmar.2009.08.001

[B40] Sanchez G. (2013). *PLS Path Modeling with R.* Berkeley, CA: Trowchez Editions.

[B41] Shedletsky L. J. Aitken J. E. (2001). The paradoxes of online academic work. *Commun. Educ.* 50 206–217. 10.1080/03634520109379248

[B42] Tarafdar M. Pullins E. B. Ragu-Nathan T. (2015). Technostress: negative effect on performance and possible mitigations. *Inform. Syst.* 25 103–132. 10.1111/isj.12042

[B43] Tarafdar M. Tu Q. Ragu-Nathan T. (2010). Impact of technostress on end-user satisfaction and performance. *J. Manag. Inform. Syst.* 27 303–334. 10.2753/MIS0742-1222270311

[B44] Tarafdar M. Tu Q. Ragu-Nathan T. Ragu-Nathan B. S. (2011). Crossing to the dark side: examining creators, outcomes, and inhibitors of technostress. *Commun. ACM* 54 113–120. 10.1145/1995376.1995403

[B45] Tenenhaus M. Amato S. Esposito Vinzi V. (2004). “A global goodness-of-fit index for PLS structural equation modelling,” in *Proceedings of the XLII SIS Scientific Meeting*, (Padova: CLEUP).

[B46] Wetzels M. Odekerken-Schröder G. Van Oppen C. (2009). Using PLS path modeling for assessing hierarchical construct models: guidelines and empirical illustration. *MIS Q.* 30 177–195. 10.2307/20650284

[B47] Willaby H. W. Costa D. S. Burns B. D. MacCann C. Roberts R. D. (2015). Testing complex models with small sample sizes: a historical overview and empirical demonstration of what partial least squares (PLS) can offer differential psychology. *Pers. Individ. Differ.* 84 73–78. 10.1016/j.paid.2014.09.008

