# Supplementary material for: Technostress Among University Teachers in Higher Education: A Study Using Multidimensional Person-Environment Misfit Theory
Source: Front Psychol. 2019 Aug 6;10:1791. doi: 10.3389/fpsyg.2019.01791 (PMC6691142; doi:10.3389/fpsyg.2019.01791)
Supplement: Supplementary file 1 [file Table_1.DOCX]

**Appendix A. Survey instrument for technostress in higher education.**

| Constructs | Items | Sources |
| --- | --- | --- |
| ADO | 1. I find it difficult to meet the high demands of school policies regarding the use of ICTs at work. | Chuang, Shen, & Judge (2016);  Edwards, Caplan, & Harrison (1998);  Jansen, & Kristof-Brown (2006). |
|  | 1. I find it difficult to effectively implement school policies regarding the use of ICTs at work. |  |
|  | 1. My current capability is insufficient to implementing school policies regarding the use of ICTs at work. |  |
|  | 1. My current skillset is insufficient for the successful implementation of school policies regarding the use of ICTs at work. |  |
|  | 1. I find it hard to adjust my current work pattern so as to comply with school policies regarding the use of ICTs at work. |  |
|  | 1. I do not have sufficient time and effort to effectively carry out school policies regarding ICT use at work. * |  |
|  | 1. I have to work harder so as to effectively implement school policies regarding the use of ICT at work. * |  |
|  | 1. I have to work faster so as to effectively implement school policies regarding ICT use at work. * |  |
| NSO | 1. My school does not provide me with sufficient professional training to effectively use ICTs at work. |  |
|  | 1. My school does not provide me with sufficient incentives to effectively use ICTs at work. |  |
|  | 1. The professional training provided by my school is not very relevant for the effective use of ICTs at work. |  |
|  | 1. I do not have a culture in my school that encourages the use of innovative tools such as ICTs at work. |  |
|  | 1. My school does not provide me with sufficient support to effectively use ICTs at work. * |  |
| ADT | 1. I feel pressured to effectively use ICTs at work. |  |
|  | 1. I find it difficult to effectively use ICTs due to my limited investment of time and effort. |  |
|  | 1. I find it difficult to cope with the high demands of ICTs with my current capability. |  |
|  | 1. I find it difficult to catch up with the rapid changes of ICTs with my current skillset. |  |
|  | 1. I find it difficult to change my current work style to meet the requirements of ICTs. * |  |
|  | 1. I am obliged to work harder because of ICTs. * |  |
|  | 1. I am obliged to work faster because of ICTs. * |  |
| NST | 1. The ICTs in my school are not effective in helping me increase my productivity at work. |  |
|  | 1. The ICTs in my school are not very relevant for the improvement of my work. |  |
|  | 1. I am irritated by the vast variety of ICTs that are utilized in my school. |  |
|  | 1. The various ICTs complicate my decision-making process at work. |  |
|  | 1. I am annoyed by the excessive use of ICTs in my school. |  |
|  | 1. I am not comfortable with the pervasive invasion of ICTs in all aspects of my work. * |  |
|  | 1. The heavy reliance on ICTs in my school disrupts my normal work pattern. * |  |
| PPF | 1. I do not have sufficient support from my colleagues for the use of ICTs at work. |  |
|  | 1. My colleagues are not encouraging with regard to the innovative use of ICTs at work. |  |
|  | 1. I do not have a team to collaborate with so as to figure out an effective way to use ICTs at work. |  |
|  | 1. I often feel that I am alone in exploring the innovative use of ICTs at work. |  |
| JP | 1. The ICTs in my school improve the quality of my work. | Tarafdar, Tu, & Ragu-Nathan (2010) |
|  | 1. The ICTs in my school enhance my work productivity. |  |
|  | 1. The ICTs in my school allow me to perform my work duties with convenience. |  |
|  | 1. The ICTs in my school enable me to accomplish more work than would otherwise be possible. |  |
|  | 1. The ICTs in my school enable me to try out innovative ideas. |  |
|  | 1. The ICTs in my school enable me to perform my work duties in innovative ways. |  |

*Note*. * Items were removed after factor analyses; ADO=Abilities-demands misfit (P-O misfit); NSO=Needs-supplies misfit (P-O misfit); ADT=Abilities-demands misfit (P-T misfit); NST=Needs-supplies (P-T misfit); PPF=Person-people misfit (P-P misfit); JP=Job performance.

**Appendix B. Criteria of performing EFA and CFA for the multidimensional P-E misfit scale of technostress and related results.**

Exploratory factor analysis (EFA) was performed through the following procedures:

1. Bartlett’s (1950) test of sphericity and Kaiser-Meyer-Olkin (KMO) measure of sampling adequacy (Kaiser & Rice, 1974) were used to assess the factorability of correlation matrices.
2. Principal component analysis (PCA) with varimax rotation (Hair et al., 2010) was conducted on the instrument to examine its convergent and construct validity. The inclusion/exclusion of items followed three criteria (Floyd & Widaman, 1995; Tabachnick & Fidell, 2001): (a) factor loadings should be larger than 0.40; (b) items of one factor should have no or minimal cross-loadings with other factors; and (c) there should be conceptual consistency among items loaded on a shared factor. Moreover, three criteria (Noar, 2003; Worthington & Whittaker, 2006) were used to decide the number of factors of the instrument: (a) eigenvalues>1, (b) scree test, and (c) conceptual interpretability of identified factors.

Subsequently, CFA was conducted to verify the factor structure generated by EFA. Goodness of fit was examined using following criteria (Hu & Bentler, 1999; MacCallum et al., 1996; Wheaton et al., 1977):

1. The normed chi-square (χ^2^/df) is expected to range from 2.0 to 5.0.
2. The comparative fit index (CFI) is expected to be ≥0.90;
3. The normed fit index (NFI) should be ≥0.90; and
4. The root mean square error of approximation (RMSEA) is expected to be ≤0.08.

**Results of EFA and CFA of the multidimensional P-E misfit technostress scale**

|  |  |  | Factor* | | | | |  |  |
| --- | --- | --- | --- | --- | --- | --- | --- | --- | --- |
|  | Cronbach’s alpha |  | 1 | 2 | 3 | 4 | 5 | *M* | *SD* |
| Abilities-demands misfit  (in P-O misfit) | 0.90 | ADO1 | 0.76 |  |  |  |  | 2.83 | 1.11 |
|  |  | ADO2 | 0.71 |  |  |  |  | 2.87 | 1.14 |
|  |  | ADO3 | 0.73 |  |  |  |  | 2.67 | 1.15 |
|  |  | ADO4 | 0.77 |  |  |  |  | 2.84 | 1.16 |
|  |  | ADO5 | 0.75 |  |  |  |  | 2.85 | 1.13 |
| Needs-supplies misfit  (in P-O misfit) | 0.89 | NSO1 |  | 0.79 |  |  |  | 3.64 | 1.14 |
|  |  | NSO2 |  | 0.88 |  |  |  | 3.85 | 1.09 |
|  |  | NSO3 |  | 0.83 |  |  |  | 3.68 | 1.10 |
|  |  | NSO4 |  | 0.84 |  |  |  | 3.83 | 1.08 |
| Needs-supplies misfit  (in P-T misfit) | 0.86 | NST1 |  |  | 0.75 |  |  | 2.82 | 1.15 |
|  |  | NST2 |  |  | 0.78 |  |  | 2.69 | 1.17 |
|  |  | NST3 |  |  | 0.74 |  |  | 2.84 | 1.28 |
|  |  | NST4 |  |  | 0.63 |  |  | 2.94 | 1.23 |
|  |  | NST5 |  |  | 0.61 |  |  | 2.69 | 1.21 |
| Person-people misfit  (in P-P misfit) | 0.86 | PPF1 |  |  |  | 0.72 |  | 3.27 | 1.18 |
|  |  | PPF2 |  |  |  | 0.73 |  | 3.23 | 1.12 |
|  |  | PPF3 |  |  |  | 0.77 |  | 3.66 | 1.12 |
|  |  | PPF4 |  |  |  | 0.82 |  | 3.43 | 1.17 |
| Abilities-demands misfit  (in P-T misfit) | 0.79 | ADT1 |  |  |  |  | 0.77 | 3.48 | 1.17 |
|  |  | ADT2 |  |  |  |  | 0.69 | 3.32 | 1.20 |
|  |  | ADT3 |  |  |  |  | 0.68 | 2.86 | 1.19 |
|  |  | ADT4 |  |  |  |  | 0.65 | 2.91 | 1.21 |
| Model fit results of CFA | χ^2^*/df* =2.06, CFI=0.95, NFI=0.91, and RMSEA=0.06 | | | | | | | | |

*Note*. *There are differences between factor loadings calculated by factor analysis and PLS-SEM as they rely on different parameter estimation methods.

**References**

Bartlett, M. S. (1950). Tests of significance in factor analysis. *British Journal of Statistical Psychology*, *3*(2), 77-85.

Floyd, F. J., & Widaman, K. F. (1995). Factor analysis in the development and refinement of clinical assessment instruments. *Psychological Assessment*, *7*(3), 286-299.

Hair, J., Black, W., Babin, B., & Anderson, R. (2010). *Multirative data analysis: A global perspective*. Upper Saddle River, NJ: Pearson Prentice Hall.

Kaiser, H. F., & Rice, J. (1974). Little jiffy, mark IV. *Educational and Psychological Measurement*, *34*(1), 111-117.

MacCallum, R. C., Browne, M. W., & Sugawara, H. M. (1996). Power analysis and determination of sample size for covariance structure modeling. *Psychological Methods*, *1*(2), 130-149.

Noar, S. M. (2003). The role of structural equation modeling in scale development. *Structural Equation Modeling*, *10*(4), 622-647.

Tabachnick, B. G., & Fidell, L. S. (2001). *Using multivariate analysis*. Boston: Allyn and Bacon.

Wheaton, B., Muthen, B., Alwin, D. F., & Summers, G. F. (1977). Assessing reliability and stability in panel models. *Sociological Methodology*, *8*, 84-136.

Worthington, R. L., & Whittaker, T. A. (2006). Scale development research: A content analysis and recommendations for best practices. *The Counseling Psychologist*, *34*(6), 806-838.

**Appendix C. Cross-loadings of variables in the research model (*N*=343).**

|  | ADO | NSO | ADT | NST | PPF | JP |
| --- | --- | --- | --- | --- | --- | --- |
| Abilities-demands misfit (P-O misfit) |  |  |  |  |  |  |
| ADO1 | **0.85** | 0.19 | 0.55 | 0.53 | 0.44 | -0.22 |
| ADO2 | **0.84** | 0.27 | 0.50 | 0.65 | 0.45 | -0.25 |
| ADO3 | **0.86** | 0.24 | 0.56 | 0.57 | 0.46 | -0.27 |
| ADO4 | **0.83** | 0.25 | 0.55 | 0.51 | 0.40 | -0.22 |
| ADO5 | **0.82** | 0.20 | 0.49 | 0.56 | 0.41 | -0.33 |
| Needs-supplies misfit (P-O misfit) |  |  |  |  |  |  |
| NSO1 | 0.24 | **0.84** | 0.18 | 0.25 | 0.43 | -0.05 |
| NSO2 | 0.19 | **0.85** | 0.16 | 0.20 | 0.36 | -0.01 |
| NSO3 | 0.27 | **0.90** | 0.20 | 0.33 | 0.48 | -0.09 |
| NSO4 | 0.24 | **0.90** | 0.23 | 0.28 | 0.48 | -0.08 |
| Abilities-demands misfit (P-T misfit) |  |  |  |  |  |  |
| ADT1 | 0.39 | 0.24 | **0.72** | 0.28 | 0.31 | 0.04 |
| ADT2 | 0.45 | 0.21 | **0.77** | 0.41 | 0.29 | -0.18 |
| ADT3 | 0.53 | 0.18 | **0.82** | 0.47 | 0.39 | -0.11 |
| ADT4 | 0.60 | 0.10 | **0.82** | 0.44 | 0.31 | -0.14 |
| Needs-supplies misfit (P-T misfit) |  |  |  |  |  |  |
| NST1 | 0.43 | 0.25 | 0.40 | **0.72** | 0.22 | -0.22 |
| NST2 | 0.48 | 0.20 | 0.38 | **0.76** | 0.29 | -0.26 |
| NST3 | 0.56 | 0.26 | 0.45 | **0.87** | 0.41 | -0.39 |
| NST4 | 0.58 | 0.25 | 0.44 | **0.80** | 0.39 | -0.26 |
| NST5 | 0.61 | 0.28 | 0.40 | **0.83** | 0.41 | -0.34 |
| Person-people misfit (P-P misfit) |  |  |  |  |  |  |
| PPF1 | 0.42 | 0.45 | 0.31 | 0.35 | **0.82** | -0.08 |
| PPF2 | 0.50 | 0.38 | 0.41 | 0.47 | **0.85** | -0.21 |
| PPF3 | 0.41 | 0.47 | 0.33 | 0.30 | **0.84** | -0.03 |
| PPF4 | 0.36 | 0.40 | 0.31 | 0.31 | **0.82** | 0.01 |
| Job performance |  |  |  |  |  |  |
| JP1 | -0.28 | -0.11 | -0.12 | -0.33 | -0.08 | **0.90** |
| JP2 | -0.32 | -0.09 | -0.17 | -0.40 | -0.12 | **0.93** |
| JP3 | -0.29 | -0.07 | -0.12 | -0.35 | -0.10 | **0.91** |
| JP4 | -0.22 | -0.10 | -0.06 | -0.30 | -0.11 | **0.83** |
| JP5 | -0.27 | 0.01 | -0.11 | -0.28 | -0.07 | **0.87** |
| JP6 | -0.25 | 0.03 | -0.12 | -0.31 | -0.08 | **0.87** |

*Note.* The bold values are the loadings of each item on its latent variable in the research model.

**Appendix D. Detailed information of the bootstrap validation of the research model.**

| Hypotheses | | Original path coefficient | Mean.Boot | SD | Percentile 0.025 | Percentile 0.975 |
| --- | --- | --- | --- | --- | --- | --- |
| **H1** | **ADO -> ADT** | **0.616** | **0.615** | **0.040** | **0.541** | **0.682** |
| **H2** | **ADO -> NST** | **0.636** | **0.637** | **0.036** | **0.570** | **0.700** |
| **H3** | **ADO -> PPF** | **0.405** | **0.399** | **0.048** | **0.300** | **0.478** |
| H4 | NSO -> ADT | 0.057 | 0.056 | 0.048 | -0.040 | 0.135 |
| **H5** | **NSO -> NST** | **0.138** | **0.141** | **0.042** | **0.064** | **0.218** |
| **H6** | **NSO -> PPF** | **0.399** | **0.411** | **0.058** | **0.295** | **0.520** |
| **H7** | **ADO -> JP** | **-0.212** | **-0.216** | **0.088** | **-0.402** | **-0.067** |
| H8 | NSO -> JP | 0.023 | 0.023 | 0.063 | -0.116 | 0.155 |
| H9 | ADT -> JP | 0.137 | 0.129 | 0.069 | -0.009 | 0.275 |
| **H10** | **NST -> JP** | **-0.348** | **-0.345** | **0.073** | **-0.488** | **-0.207** |
| H11 | PPF -> JP | 0.087 | 0.084 | 0.067 | -0.046 | 0.225 |

*Note.* Three decimal places were kept in order to illustrate detailed statistical outcomes; The bold rows highlight the hypotheses that were supported in this study.
